# Complete mitochondrial genome of the Kyrghyz racerunner (*Eremias nikolskii* Bedriaga, 1905) from Kyrgyzstan

**DOI:** 10.1080/23802359.2022.2080599

**Published:** 2022-06-10

**Authors:** Xianguang Guo, Xiaopeng Huo, Jinlong Liu, Marina A. Chirikova

**Affiliations:** a Key Laboratory of Southwest China Wildlife Resources Conservation (Ministry of Education), China West Normal University, Nanchong, China; b Chengdu Institute of Biology, Chinese Academy of Sciences, Chengdu, China; c Research Institute of Safety, Environmental Protection and Technical Supervision, PetroChina Southwest Oil & Gasfield Company, Chengdu, China; d Institute of Zoology of Republic of Kazakhstan, Almaty, Kazakhstan

**Keywords:** Mitochondrial genome, monophyly, next-generation sequencing, phylogenetic tree, *Eremias*

## Abstract

The complete mitochondrial genome (mitogenome) of the Kyrghyz racerunner (*Eremias nikoskii* Bedriaga, 1905) from Kyrgyzstan was determined for the first time by next-generation sequencing. The mitogenome was 20,840 bp in length and comprised the standard set of 13 protein-coding genes (PCGs), 2 ribosomal RNA genes, 22 transfer RNA genes, and a control region. The 13 concatenated PCGs were used to implement Bayesian phylogenetic analyses together with some congeners and three representative lacertids retrieved from GenBank. The monophyly of both *Eremias* and its viviparous group was recovered in the Bayesian phylogenetic tree, while the subgenus *Pareremias* was paraphyletic with respect to *E. nikoskii*. The mitogenome of *E. nikoskii* will faciliate the research on species delimitation, molecular evolution, and phylogenetic inference in the racerunner lizards.

The Kyrghyz racerunner, *Eremias nikoskii* Bedriaga, 1905, is an oviparous species among subgenus *Aspidorhinus* (Barabanov 2009) in genus *Eremias*. This species occurs in the mountain ranges around the Fergana valley in eastern Uzbekistan, Kyrgyzstan, and northern Tajikistan, and prefers mountain ravines and the valleys of mountain rivers, in rocky areas with thin grass and bush cover up to about 1000–3000 m above sea level (Szczerbak 2003). To date, little is known about its genetic affinities with congeners albeit with limited understanding of phylogeny of the racerunner (*Eremias*) lizards (Guo et al. 2011; Khan et al. 2021).

In this study, we reported the whole mitogenome of *E. nikolskii* for the first time, with voucher number Guo4717. This specimen was collected from westward of Kazarman village (41.38750˚N, 73.93999˚E, 1332 meters above sea level), Dzhalalabad region in Kyrgyzstan on 28 August 2014. Its liver tissue was dissected from the euthanized lizard, fixed with 95% ethanol, and stored at −20 °C in the Chengdu Institute of Biology, Chinese Academy of Sciences (contact person: Xianguang Guo, E-mail: guoxg@cib.ac.cn). The CIB Animal Care and Use Committee approved all procedures.

Total genomic DNA was extracted from the liver tissue in the Genepioneer Biotechnologies Co. Ltd. (Nanjing, China) for 150 bp paired-end (PE150) library construction as well as sequencing through the Illumina NovaSeq (Illumina, USA). The raw data were processed with fastp v0.20.0 (Chen et al. 2018) by trimming adapters and primers, and filtering low quality reads. Assembly of clean data was performed using SPAdes v3.10.1 (Bankevich et al. 2012). Subsequently, we took a similar strategy to that in Wang et al. (2021) to obtain the complete mitogenome. The mitogenome was preliminarily annotated with MITOS Web Server (http://mitos2.bioinf.uni-leipzig.de; Bernt et al. 2013). Twenty-two tRNA genes were confirmed by using the software tRNA scan-SE (Lowe and Chan 2016). The nucleotide composition was estimated in MEGA v7.0 (Kumar et al. 2016).

The complete mitogenome of *E. nikoskii* was 20,840 bp in length, which was composed of 28.00% T, 28.01% C, 30.54% A, 13.45% G. A total of 37 genes were obtained including 13 protein-coding genes (PCGs), 22 transfer RNA (tRNAs), 2 ribosomal RNA gens, and a control region (CR or D-loop). The gene content, order, and composition exhibited a typical vertebrate mtDNA feature. Most genes were distributed on H-strand, with exception to *ND6* gene and eight tRNAs (*tRNA-Glu, Ala, Asn, Cys, Tyr, Ser^[UGA]^, Gln,* and *Pro*). In the 13 PCGs, the shortest one was *ATP8* gene (162 bp) and the longest one was *ND5* (1824 bp). Only *COX1* gene used GTG as a start codon, while the other PCGs used ATG. Five PCGs (*ND1*, *ATP8*, *ATP6*, *ND4L*, *ND5*) used TAA as stop codon; five PCGs (*ND2*, *COX2*, *COX3*, *ND3*, *ND4*) used T; two PCGs (*COX1*, *ND6*) used AGG; *Cytb* used TAG. In addition, 12S rRNA, 16S rRNA, and CR were 951, 1545, and 5436 bp, respectively.

Phylogenetic trees were inferred from the concatenated PCGs of *Eremias* spp. and other representative lacertids (Lacertidae) retrieved from GenBank. Bayesian analyses were conducted using MrBayes v3.2.7a (Ronquist et al. 2012) with the GTR + G + I substitution model. A 50% majority-rule consensus tree was assessed by combining the sampled trees from two independent runs after a 30% burn-in phase. Clade support was assessed by posterior probability (PP). As shown in Figure 1, the monophyly of both genus *Eremias* and its viviparous group was recovered with strong support, which is consistent with previous studies (Guo et al. 2011; Orlova et al. 2017; Liu et al. 2021). *Eremias nikoskii* was inferred as the sister taxon to the viviparous group with strong support (PP = 0.95); this challenged monophyly of the subgenus *Pareremias* (Arnold 1986; Guo et al. 2011). The mitogenome of *E. nikoskii* will facilitate the research on species delimitation, molecular evolution, and phylogenetic inference in the racerunner lizards.

**Figure 1.**
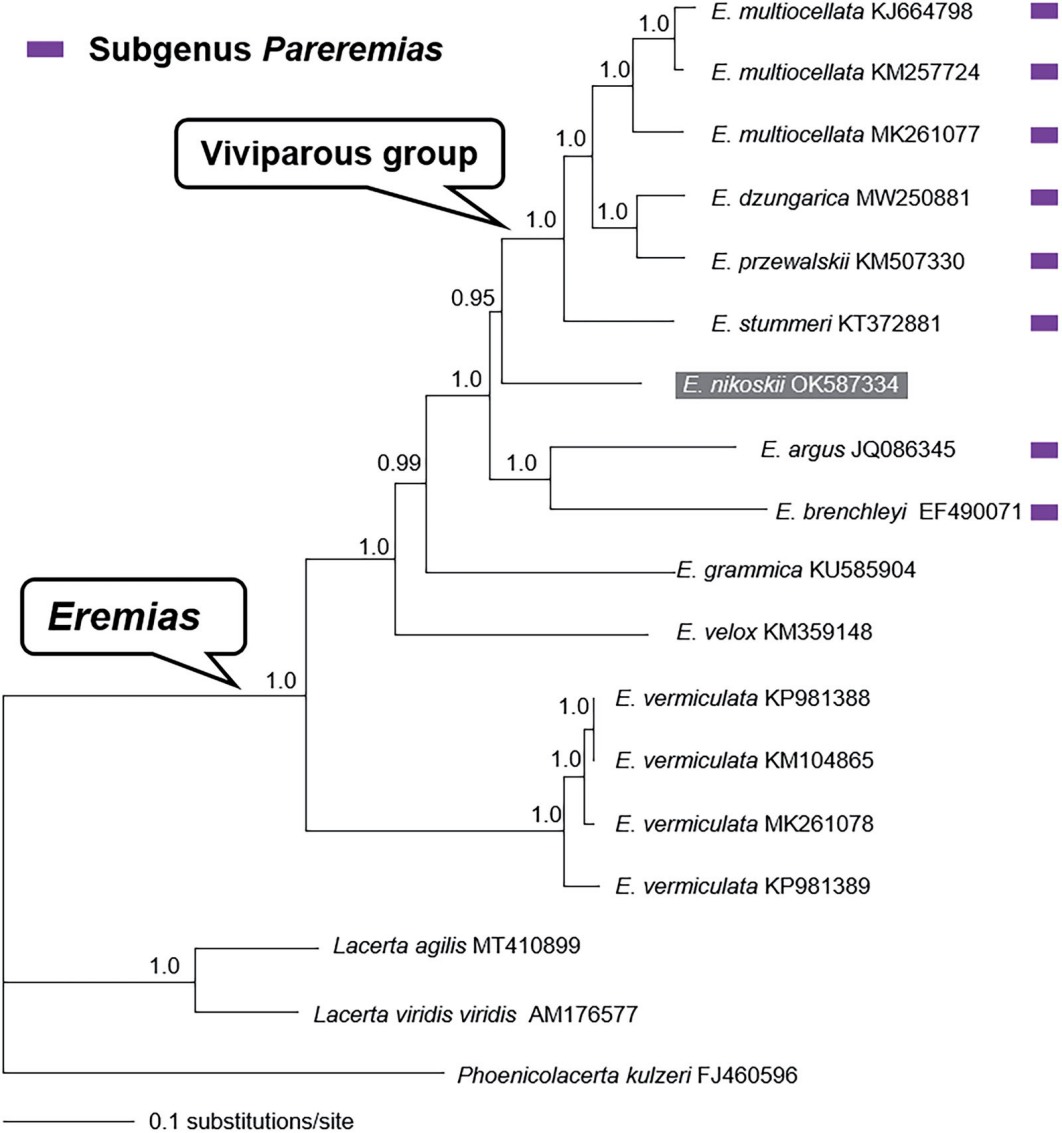
Bayesian phylogenetic tree inferred from the 13 concatenated PCGs with GTR + G + I substitution model. Node numbers show Bayesian posterior probabilities. GenBank accession numbers are given with species names, and the phylogenetic position of *E. nikolskii* is highlighted.

## Data Availability

The genome sequence data that support the findings of this study are openly available in GenBank of NCBI at (https://www.ncbi.nlm.nih.gov/nuccore/OK587334) under the accession number OK587334. The associated BioProject, SRA, and Bio-Sample numbers are PRJNA773219, SRR16509497, and SAMN22448698, respectively.
